# Lactate Dehydrogenase Inhibitors Suppress *Borrelia burgdorferi* Growth In Vitro

**DOI:** 10.3390/pathogens12070962

**Published:** 2023-07-22

**Authors:** Adam Lynch, Patrick Pearson, Sergey N. Savinov, Andrew Y. Li, Stephen M. Rich

**Affiliations:** 1 Department of Microbiology, University of Massachusetts, Amherst, MA 01003, USA; aclynch@umass.edu (A.L.); pbpearson@umass.edu (P.P.); 2 Department of Biochemistry, University of Massachusetts, Amherst, MA 01003, USA; sergeysavinov2@gmail.com; 3 Invasive Insect Biocontrol & Behavior Laboratory, USDA-ARS, Beltsville, MD 20705, USA; andrew.li@usda.gov

**Keywords:** *Borrelia burgdorferi*, LDH inhibitors, lactate dehydrogenase, gossypol

## Abstract

*Borrelia burgdorferi*, the causative agent of Lyme disease, has a highly reduced genome and relies heavily on glycolysis for carbon metabolism. As such, established inhibitors of lactate dehydrogenase (LDH) were evaluated in cultures to determine the extent of their impacts on *B. burgdorferi* growth. Both racemic and enantiopure (AT-101) gossypol, as well as oxamate, galloflavin, and stiripentol, caused the dose-dependent suppression of *B. burgdorferi* growth in vitro. Racemic gossypol and AT-101 were shown to fully inhibit spirochetal growth at concentrations of 70.5 and 187.5 μM, respectively. Differences between racemic gossypol and AT-101 efficacy may indicate that the dextrorotatory enantiomer of gossypol is a more effective inhibitor of *B. burgdorferi* growth than the levorotatory enantiomer. As a whole, LDH inhibition appears to be a promising mechanism for suppressing *Borrelia* growth, particularly with bulky LDH inhibitors like gossypol.

## 1. Introduction

Lyme disease is the most common zoonotic illness reported in North America. The range of the disease’s primary arthropod vector *Ixodes scapularis* is expanding, potentially increasing the risk of infection in much of the United States [1]. Current treatment strategies are effective in most cases, but as many as 17% of patients may remain partially symptomatic one year after treatment [2]. Chemoprophylactic doxycycline dosing has been shown to effectively reduce the risk of Lyme disease manifestation following tick bite exposure [3]. However, prophylaxis may be problematic due to potentially adverse side effects of antibiotic treatment, the relatively low rate of disease manifestation after single tick bites, and the general risks associated with antibiotic overuse [4,5]. As such, expanding the arsenal of existing Lyme disease management tools is undoubtedly beneficial. 

Lyme disease’s causative agent, *Borrelia burgdorferi*, has a highly reduced genome and is therefore relegated to a specialized metabolism. *B. burgdorferi* lacks a tricarboxylic (TCA) cycle, cannot conduct oxidative phosphorylation, and has no pathways for carbohydrate, amino acid, or lipid biosynthesis [6]. Instead, glycolysis is the sole mechanism for *B. burgdorferi*’s ATP production. Furthermore, because it lacks both pyruvate dehydrogenase and pyruvate oxygenase, the sole means by which *B. burgdorferi* can use pyruvate is by conversion to lactate via the enzyme, lactate dehydrogenase (LDH) [7]. Thus, LDH is an essential metabolic linchpin for *B. burgdorferi*, and its inhibition could serve as an effective method for mitigating cell growth. This atypical metabolic circumstance presents an opportunity to target the spirochete with minimal collateral effects on the host.

LDH inhibitors have been widely suggested for the treatment of various cancers and apicomplexan infections [8,9,10,11]. In humans, there are a multitude of LDH types consisting of different tissue-associated combinations of two subunits, with the most prominent combinations being classified as LDHA (skeletal LDH) and LDHB (heart LDH) [12]. Both LDHA and LDHB play roles in the Warburg effect, the phenomenon of increased levels of anaerobic glycolysis observed in cancer cells compared to noncancerous cells [12,13]. Under these conditions, LDH becomes an indispensable resource to cells and its inhibition is shown to reduce tumor growth [14]. Compared to current anticancer chemotherapies, selective LDH inhibition has few side effects: complete LDHA deficiency in humans is almost universally asymptomatic, further increasing the attractiveness of anti-LDH drugs for cancer chemotherapy [15]. Importantly, commercially available LDH inhibitors show efficacy against the LDH of eukaryotic microorganisms such as those possessed by *Cryptosporidium parvum* [9], as well as those for several species of tickborne pathogens within the genus *Babesia* [16,17]. 

As such, we propose the repurposing of existing LDH inhibitors to impede *B. burgdorferi* growth. Here, we evaluate the effects of commercially available LDH inhibitors on the in vitro growth of *B. burgdorferi* using a standardized growth assay. The LDH inhibitors that we used cover a variety of mechanisms: competitive in respect to pyruvate (oxamate), competitive in respect to NADH (gossypol, AT-101, GSK2837808A), competitive with both NADH and pyruvate (NHI-2, galloflavin, FX-11), and noncompetitive inhibitors (stiripentol, isosafrole) [10,18,19,20,21,22,23,24,25]. To a lesser extent, gossypol also shows noncompetitive inhibition of pyruvate [26]. The understanding of these mechanisms, in conjunction with in vitro performance, will guide future in vivo evaluations of these compounds and, ultimately, the development of a new generation of tools for managing Lyme disease. 

## 2. Materials and Methods

### 2.1. Acquisition of LDH Inhibitors

All LDH inhibitors (AT-101 (Catalog #SML0433, ≥98%), FX-11 (Catalog #427218, ≥96%), galloflavin (Catalog #SML0776, ≥95%), gossypol (Catalog #G8761, ≥95%), GSK2837808A (Catalog #5.33660, ≥95%), isosafrole (Catalog #329606, ≥100%), sodium oxamate (Catalog #02751, ≥98%), stiripentol (Catalog #S6826, ≥98%), and NHI-2 (Catalog #SML1463, ≥98%)) were purchased from Sigma-Aldrich (Burlington, MA, USA). 

### 2.2. Growth Inhibition Assays

Our growth inhibition methodology was adapted from Caol et al. [27]. A uniform *B. burgdorferi* stock was created and used for all assays. *B. burgdorferi* strain B31 clone 5A2 was acquired from BEI Resources (Manassas, VA, USA) as a frozen glycerol stock. A low-passage (<3) sample was incubated in closed, screw-cap vials containing 14 mL BSK-H complete media (Sigma Aldrich) at 34 °C for nine days, until cells reached a concentration of greater than 10^7^ cells/mL. Cells were manually counted utilizing darkfield microscopy and C-Chip disposable hemocytometers (INCYTO), before being diluted to 1.1 × 10^6^ cells/mL in 50 mL of BSK-H complete media. Next, 900 μL aliquots were added to cryovials, and 100 μL DMSO was added as cryoprotectant. Cells were stored at −80 °C. 

LDH inhibitors were dissolved in DMSO, added to BSK-H complete media, and then sterilized using vacuum filtration (0.22 μm PVDF filter). Oxamate is not sufficiently soluble in DMSO and was instead directly dissolved into BSK-H complete media before filtration. In-house vehicle control data indicate no significant difference in *B. burgdorferi* growth in unaltered media compared to media with concentrations of less than 0.5% DMSO (Figure S1, *p* = 0.94). LDH inhibitors were then serially diluted in BSK-H media to achieve the desired final concentrations. 

*B. burgdorferi* cells were grown from the previously created cryovials, as described above. *Borrelia* was diluted into a large single stock of 50 mL BSK-H and mixed thoroughly to ensure a homogeneous distribution of cells. Cells were taken from this working mixture and added to inhibitor–BSK solutions, achieving a final concentration of 1.0 × 10^6^ cells/mL. Following the method of Caol et al. [27], cells were then incubated at 34 °C for 72 h, before being counted manually using darkfield microscopy. Bacteriostatic concentrations were defined as any concentration after 72 h in which cell counts did not exceed the base concentration. To allow for random fluctuations in cell counts, three dilutions to 10^6^ cells/mL were conducted, and each was counted three times. The standard deviation was calculated. Assuming a normal distribution of cell counts, twice this standard deviation was added to the initial inoculum, establishing 1.33 × 10^6^ cells/mL as the cutoff point for bacteriostatic concentrations. 

Each drug concentration was tested in triplicate. In cases where strong dose-dependent responses were present, experiments were repeated with alterations to drug dosage to better determine the half-maximal inhibitory concentration (IC_50_). The minimum inhibitory concentration (MIC) was defined as the smallest dosage required to fully inhibit bacterial growth and was calculated as the midpoint between the lowest bacteriostatic concentration and the highest non-bacteriostatic concentration. For example, in a case where no cell growth was seen at a concentration of 100 uM of an LDH inhibitor and the next highest dosage was 50 μM of a given inhibitor, we would estimate the MIC as 75 μM. 

### 2.3. Minimum Bactericidal Concentration (MBC) Assays

Our MBC determination methodology was adapted from Sicklinger et al. [28]. In instances where no bacterial growth was evident, a 25 μL sample was taken from all replicates with inhibitor concentrations greater than or equal to the MIC. Each sample was added to 1 mL of inhibitor-free BSK-H. Cells were then incubated for two weeks at 34 °C, after which they were observed for living spirochetes under a darkfield microscope. If no motile spirochetes were visible, the concentration of the LDH inhibitor was considered bactericidal. The reported MBCs are the mode values at which concentrations were considered bactericidal. 

### 2.4. Data Analysis

Analyses were conducted using GraphPad Prism 8.3.2 for Windows (GraphPad Software, San Diego, CA, USA. To account for variations in cell growth between replicate experiments, growth numbers were normalized within each experiment, compiled, and fitted with a nonlinear regression line. Normalization was relative to the average cell growth of each experiment’s vehicle control group, with the control quantified as 100% and instances with no growth (≤100 visible cells) quantified as 0%. IC_50_ values were determined using dose-dependent normalized response curves.

## 3. Results

The impacts of LDH inhibitors on *Borrelia* growth varied greatly between compounds (Table 1). Of all compounds, racemic gossypol was shown to have the lowest IC_50_ and MIC values: 14 μM and 70.5 μM, respectively. Notably, the levorotatory enantiomer of gossypol, AT-101, demonstrated a significantly lower impact than the racemic mixture with IC_50_ and MIC values of 50 μM and 187.5 μM, respectively (Figure S2, *p* = 0.0026). AT-101 and racemic gossypol both demonstrated bactericidal properties: AT-101 was found to have an MBC value of 250 μM, while gossypol displayed an MBC value of 150 μM. 

**Figure 1:**
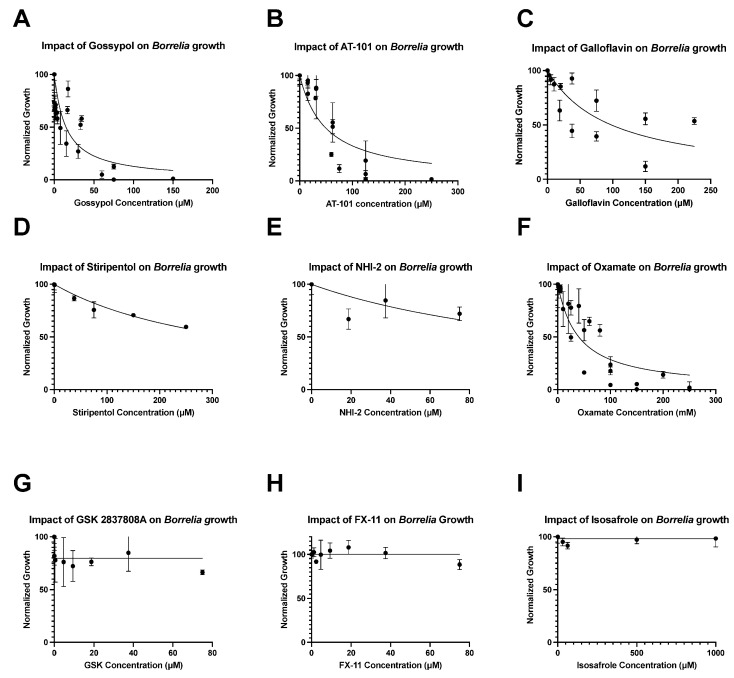
Impact of LDH inhibitors on *Borrelia burgdorferi* growth. The inhibitors, gossypol (**A**), AT-101 (**B**), stiripentol (**C**), galloflavin (**D**), oxamate (**E**), and NHI-2 (**F**), are fitted with normalized dose–response curves. The inhibitors, GSK 2837808A (**G**), FX-11 (**H**), and isosafrole (**I**), are more appropriately fitted with horizontal lines; responses are not dose-dependent. Each point reflects the mean of three replicates for a single drug concentration. Cells were counted manually using darkfield microscopy.

Galloflavin and stiripentol also showed strong dose-dependent effects on *Borrelia* growth, with IC_50_s of 98 μM and 350 μM, respectively, but did not yield MIC or MBC values at the tested concentrations (Figure 1C,D). Similarly, while NHI-2 had a noticeable impact on *Borrelia* growth at high concentrations with an IC_50_ of 150 μM, the overall dose dependence was low: data were highly variant, and the growth curve had an R^2^ value of only 0.32 (Figure 1E). 

Oxamate had the highest calculable IC_50_ of all inhibitors tested, with an IC_50_ of 38 mM and a MIC of 125 mM (Figure 1F). At 200 mM, oxamate was bactericidal to cells in all instances, with no motile spirochetes in cultures observed in any instance after re-incubation into oxamate-free media (Table 1).

The inhibitors, isosafrole, FX-11, and GSK2837808A, exhibited little to no effect on Borrelia growth in vitro (Figure 1G–I). Therefore, no MIC, MBC, or IC_50_ could be determined for these compounds, and no significant inhibitory effect was seen, even at the highest concentrations of the inhibitors (Table 1). 

## 4. Discussion

LDH inhibitors have been tested against cancer cells and apicomplexans but are less well studied against bacteria [8,10]. Furthermore, this is the first instance that LDH inhibitors have been tested on *Borrelia burgdorferi*. As such, a range of commercially available LDH inhibitors with various mechanisms of action and origins were tested on *Borrelia* in culture. Of these inhibitors, gossypol, AT-101, and oxamate substantially impacted *B. burgdorferi* growth in vitro and represent promising candidates against *Borrelia* infections in vivo. While oxamate’s IC_50_ concentration is easy to achieve in vitro due to its high solubility in water, it requires a dose several orders of magnitude higher than other active compounds. Nonetheless, it should be noted that these concentrations are comparable to those found for the treatment of human cancer cells [29]. As such, it may be possible that oxamate is more effective when used in tandem with other compounds, rather than as a sole treatment option, as is demonstrated in multiple anti-cancer studies [8,22,30]. 

In terms of bacteriostatic inhibition, racemic gossypol was shown to have a lower MIC than AT-101, the levorotatory enantiomer of gossypol. Prior studies have shown the variance in the efficacy of the gossypol enantiomers, even when used against human LDH subtypes: levorotatory gossypol more effectively inhibits human LDHA [31], while *dextrorotatory* gossypol more effectively inhibits human placental LDH [32]. The increased efficacy of racemic gossypol relative to AT-101 suggests that dextrorotatory gossypol is the more effective enantiomer in *Borrelia* growth inhibition. This differential is promising for dextrorotatory gossypol’s potential as a treatment option: gossypol toxicity is primarily caused by the levorotatory enantiomer [24,33]. Likewise, though gossypol has been tested as a male infertility agent, levorotatory gossypol is the primary cause of antifertility activity [34]. Thus, focusing on the dextrorotatory enantiomer may significantly reduce concerns of both toxicity and infertility. 

Although both FX-11 and GSK2837808A are shown to be effective on human cancer cells in vitro at nanomolar and micromolar levels [10,25], and isosafrole is shown to be a potent human LDHA inhibitor [22], these agents had no effect on *Borrelia* cells. Isosafrole’s exact mechanism and site of interaction remains unknown, and the relatively large size (MW = 649.6) and branching topology of NADH-competitive GSK2837808A likely compromises its scope in targeting LDH types from evolutionarily distant organisms [35]. However, the lack of activity of gossypol-like FX-11 is more intriguing and may offer some insight into the further design of potent *B. burgdorferi* LDH (*Bb*LDH) modulators. FX-11 and gossypol are expected to differ in charge under physiological conditions, since the former is a carboxylic acid; however, protein sequence alignment attests to the high conservation of charged residues in the vicinity of the substrate-binding site in both human LDHA and *Bb*LDH (Figure S3). Hence, this lack of activity cannot be readily attributed to a difference in charge but instead points to size as the characteristic that causes the highly different activities of these structurally related compounds. The fact that racemic gossypol is the most active of all tested agents, while its smaller variant FX-11 lacks anti-*Borrelia* activity altogether is counterintuitive, since the latter is expected to fit where the larger counterpart binds. 

The disparity between gossypol and FX-11 efficacy may be explained by the well-established structural dynamics of the active site in LDH enzymes. The LDH active site mobile loop can adapt to either open and closed states depending on the substrate and cofactor association status [36]. Unlike the more compact inhibitors, FX-11 and galloflavin, which are competitive with both pyruvate and NADH [37], bulky and rigid gossypol does not fit into the compact pocket formed upon mobile loop closure and is therefore expected to bind to the ‘open’ forms of LDH (Figure S4). Gossypol is only competitive with NADH and non-competitive with pyruvate [26], suggesting that it targets a conformational state where the substrate subsite is not yet fully organized. Consequently, we hypothesize that gossypol associates with an open form of *Bb*LDH and relies on its bulk to reach the level of affinity that translates into observed spirochetal growth suppression, whereas smaller substrate-competitive inhibitors target the closed form of LDHA. The minimal activities of such inhibitors against *B. burgdorferi* suggest that mobile loop closure may amplify differences between rather conserved active sites in human LDHA and *Bb*LDH, pointing to the open conformation for development of effective chemotherapeutics against *Borrelia*. These differences are also reflected in the observation that the two enzymes display distinct preferences for the two enantiomers of gossypol. While human LDHA is more sensitive to its levorotatory form, *Bb*LDH apparently is inhibited more effectively by dextrorotatory gossypol. These observations will guide modeling studies to develop more effective anti-*Borrelia* chemotherapeutics. 

A potential limitation of these conclusions is the possibility that growth reduction was not caused by LDH inhibition, but was instead the result of off-target effects. This is a valid concern, somewhat mitigated by the fact that several mechanistically distinct LDH inhibitors (gossypol, oxamate, and galloflavin) displayed dose-dependent (albeit variable) growth suppression effects. Nonetheless, currently available LDH inhibitors have notable off-target effects. Oxamate, for example, is a pyruvate analog [38]. Due to this, oxamate can inhibit aspartate aminotransferase, impact the function of malate dehydrogenase, and otherwise disrupt cellular processes requiring pyruvate [39]. The effects might be concerning if focusing on other organisms, but *B. burgdorferi*’s reduced genome leaves only LDH as a means of utilizing pyruvate, making off-target effects significantly less likely [7]. As such, the presence of dose-dependent growth responses to oxamate addition suggests that LDH inhibition alone is sufficient to reduce *B. burgdorferi* growth.

Concerns regarding off-target effects of gossypol are less easily dismissed. In addition to LDH inhibition, gossypol is known to cause oxidative damage to cells [40,41]. *B. burgdorferi* has a repertoire of genes to protect itself from common reactive oxygen species but remains susceptible to some forms of oxidative damage [42]. One possible way to test whether gossypol inhibits growth through oxidative damage, LDH inhibition, or some combination thereof could be to evaluate the effects of gossypol on *Borrelia turicatae*. A distant relative within the *Borrelia* genus, *B. turicatae* has recently been found to be substantially more resistant to oxidative damage than *B. burgdorferi* [43]. Overall, *Borrelia* species have a similarly reduced genome to *B. burgdorferi*, lacking many metabolic pathways and likely relying on the same mechanisms for energy production [44]. If the addition of gossypol to *B. turicatae* culture reduces bacterial growth to a comparable extent, it may indicate that the reduction in *B. burgdorferi* growth is at least partly due to LDH inhibition. 

Taken together, the efficacy of select LDH inhibitors is an encouraging indicator that supports the pursuit of a *Bb*LDH-specific inhibitor as an asset for the management of *Borrelia* infection. To achieve this, research establishing the efficacy of gossypol against *B. burgdorferi* infection in animal models is warranted. While gossypol has shown toxicity in humans, the asymptomatic nature of total human LDH deficiency suggests that selective LDH inhibition alone should have minimal side effects [15]. With the theoretical merits of LDH inhibition in mind, in conjunction with the practical results demonstrated here, future steps should involve the characterization of the crystal structure of *Bb*LDH as an invaluable resource for the discovery of *Bb*LDH specific inhibitors. As a whole, LDH inhibition is a promising mechanism for *Borrelia* control, particularly with bulky LDH inhibitors like gossypol. 

## Figures and Tables

**Table 1:** Growth inhibition according to LDH inhibitor.

Compound	IC_50_	MIC	MBC
Gossypol (+/−)	14 µM	70.5 µM	150 µM
AT-101	50 µM	187.5 µM	250 µM
Galloflavin	98 µM	-	-
NHI-2	150 µM	-	-
Stiripentol	350 µM	-	-
Oxamate	38 mM	125 mM	200 mM
FX-11	-	-	-
Isosafrole	-	-	-
GSK2837808A	-	-	-

IC_50_ values were determined using GraphPad prism 8.32, via normalized dose-dependent response curves, and reported to two significant figures. A “-” indicates that the metric does not occur within the tested range of compounds.

## Data Availability

Data are available upon request. This article reports the results of research only. Mention of a proprietary product does not constitute an endorsement or a recommendation by the authors or USDA for its use. The USDA is an equal opportunity provider and employer.

## Supplementary Materials

The following supporting information can be downloaded at: https://www.mdpi.com/article/10.3390/pathogens12070962/s1, Figure S1: Correlation between cell counts grown in DMSO versus unaltered media. Mean cell counts of DMSO controls to non-DMSO controls were compared between paired experiments. There is no significant difference between vehicle control groups treated with DMSO and untreated groups. (GraphPad Prism 8.3.1: Paired *t*-test. t = 0.076, df = 8, *p* = 0.94, r = 0.9974). Figure S2: Comparative impacts of racemic gossypol and AT-101 on *B. burgdorferi*. Cells were counted manually using darkfield microscopy. Each point reflects the mean of three replicates for a single concentration. (Graphpad Prism 8.3.2: Comparison of fits, *p* ≤ 0.0001, r2 values of 0.52 and 0.77 for gossypol and AT-101 curves, respectively). Figure S3: A pairwise sequence alignment of *Bb*LDH and human LDHA. Black shading indicates identity, while gray indicates similarity. Overall identity is 37%. This is increased to 100% at the active site and substrate binding sites, highlighted with red and blue text, respectively. Sequence alignment and similarity determination was conducted utilizing the available FASTA from the Uniprot Universal Protein Resource (*Bb*LDH AC:O51114, Human LDHA AC: P00338) with a BLOSUM50 matrix using T-Coffee [45,46]. Figure S4: (A). Comparative structures of closed- and open-form LDH complexed with gossypol. The structure of *Borrelia burgdorferi* LDH was generated via homology modeling with Prime/Schrödinger (Schrödinger Release 2020-1: Prime, Schrödinger, LLC, New York, NY, USA, 2020) using the available LDH structures, 1LDN (*Geobacillus stearothermophilus* LDH, DOI: 10.2210/pdb1ldn/pdb) and 3WSW (*Enterococcus mundtii* LDH, PDB DOI: 10.2210/pdb3WSW/pdbB) from the RCSB protein databank for open- and closed-form LDH, respectively. Structures were chosen based on their overall homology, active site identity, and ligand presence. The LDH mobile loop is highlighted in red, while the rest of the protein is in white. The ligands (NAD and OXM), shown in stick rendering, were retained from the template structures (carbon = green, oxygen = red, nitrogen = blue, phosphorus = purple, heteroatom hydrogen = white). (B). Electrostatic interactions between LDH forms and ligands. Glide/Schrödinger was used to model gossypol access to the open and closed loop of *Bb*LDH models: (i) Electrostatic surfaces of the open and closed form models (negative charge = red, positive charge = blue, neutral = grey) with stick rendering of the ligands (NAD and OXM); (ii) conformations of NAD ligand retained from the homology template and gossypol (displaying the best docking score to the “open” model of *Bb*LDH), rendered as electrostatic surfaces; (iii) the same conformations of NAD and gossypol, rendered as stick models.

## References

[1] Eddens T., Kaplan D.J., Anderson A.J.M., Nowalk A.J., Campfield B.T., Eddens T. (2018). Insights from the Geographic Spread of the Lyme Disease Epidemic. Clin. Infect. Dis..

[2] Wormser G.P., Ramanathan R., Nowakowski J., McKenna D., Holmgren D., Visintainer P., Dornbush R., Singh B., Nadelman R.B. (2003). Duration of Antibiotic Therapy for Early Lyme Disease: A Randomized, Double-Blind, Placebo-Controlled Trial. Ann. Intern. Med..

[3] Nadelman R.B., Nowakowski J., Fish D., Falco R.C., Freeman K., McKenna D., Welch P., Marcus R., Agüero-Rosenfeld M.E., Dennis D.T. (2001). Prophylaxis with Single-Dose Doxycycline for the Prevention of Lyme Disease after an *Ixodes scapularis* Tick Bite. N. Engl. J. Med..

[4] Stanek G., Strle F. (2018). Lyme borreliosis–from tick bite to diagnosis and treatment. FEMS Microbiol. Rev..

[5] Warshafsky S., Lee D.H., Francois L.K., Nowakowski J., Nadelman R.B., Wormser G.P. (2010). Efficacy of antibiotic prophylaxis for the prevention of Lyme disease: An updated systematic review and meta-analysis. J. Antimicrob. Chemother..

[6] Zhang K., Bian J., Deng Y., Smith A., Nunez R.E., Li M.B., Pal U., Yu A.-M., Qiu W., Ealick S.E. (2016). Lyme disease spirochaete Borrelia burgdorferi does not require thiamin. Nat. Microbiol..

[7] Corona A., Schwartz I. (2015). *Borrelia burgdorferi*: Carbon Metabolism and the Tick-Mammal Enzootic Cycle. Microbiol. Spectr..

[8] Xing B.-C., Wang C., Ji F.-J., Zhang X.-B. (2018). Synergistically suppressive effects on colorectal cancer cells by combination of mTOR inhibitor and glycolysis inhibitor, Oxamate. Int. J. Clin. Exp. Pathol..

[9] Zhang H., Guo F., Zhu G. (2015). Cryptosporidium Lactate Dehydrogenase Is Associated with the Parasitophorous Vacuole Membrane and Is a Potential Target for Developing Therapeutics. PLoS Pathog..

[10] Le A., Cooper C.R., Gouw A.M., Dinavahi R., Maitra A., Deck L.M., Royer R.E., Vander Jagt D.L., Semenza G.L., Dang C.V. (2010). Inhibition of lactate dehydrogenase A induces oxidative stress and inhibits tumor progression. Proc. Natl. Acad. Sci. USA.

[11] Wu Y.W., Chik C.L., Knazek R.A. (1989). An In Vitro and In Vivo Study of Antitumor Effects of Gossypol on Human SW-13 Adrenocortical Carcinoma. Cancer Res..

[12] Urbańska K., Orzechowski A. (2019). Unappreciated Role of LDHA and LDHB to Control Apoptosis and Autophagy in Tumor Cells. Int. J. Mol. Sci..

[13] Smith B., Schafer X.L., Ambeskovic A., Spencer C.M., Land H., Munger J. (2016). Addiction to Coupling of the Warburg Effect with Glutamine Catabolism in Cancer Cells. Cell Rep..

[14] Vander Heiden M.G., Cantley L.C., Thompson C.B. (2009). Understanding the Warburg Effect: The Metabolic Requirements of Cell Proliferation. Science.

[15] Shi Y., Pinto B.M. (2014). Human Lactate Dehydrogenase A Inhibitors: A Molecular Dynamics Investigation. PLoS ONE.

[16] Vudriko P., Masatani T., Cao S., Terkawi M.A., Kamyingkird K., Mousa A.A., Moumouni P.F.A., Nishikawa Y., Xuan X. (2014). Molecular and Kinetic Characterization of *Babesia microti* Gray Strain Lactate Dehydrogenase as a Potential Drug Target. Drug Target Insights.

[17] He L., Bastos R.G., Yu L., Laughery J.M., Suarez C.E. (2022). Lactate Dehydrogenase as a Potential Therapeutic Drug Target to Control Babesia bigemina. Front. Cell. Infect. Microbiol..

[18] Granchi C., Paterni I., Rani R., Minutolo F., Kumar V., A Christopher J., Jeong Y., Kwon D., Hong S., Roychowdhury A. (2013). Small-molecule inhibitors of human LDH5. Futur. Med. Chem..

[19] Manerba M., Vettraino M., Fiume L., Di Stefano G., Sartini A., Giacomini E., Buonfiglio R., Roberti M., Recanatini M. (2011). Galloflavin (CAS 568-80-9): A Novel Inhibitor of Lactate Dehydrogenase. Chemmedchem.

[20] Sada N., Lee S., Katsu T., Otsuki T., Inoue T. (2015). Targeting LDH enzymes with a stiripentol analog to treat epilepsy. Science.

[21] Doherty J.R., Cleveland J.L. (2013). Targeting lactate metabolism for cancer therapeutics. J. Clin. Investig..

[22] Lea M.A., Guzman Y., Desbordes C. (2016). Inhibition of Growth by Combined Treatment with Inhibitors of Lactate Dehydrogenase and either Phenformin or Inhibitors of 6-Phosphofructo-2-kinase/Fructose-2,6-bisphosphatase 3. Anticancer Res..

[23] Li X., Lu W., Hu Y., Wen S., Qian C., Wu W., Huang P. (2013). Effective inhibition of nasopharyngeal carcinoma in vitro and in vivo by targeting glycolysis with oxamate. Int. J. Oncol..

[24] Wu D.-F., Yu Y.-W., Tang Z.-M., Wang M.-Z. (1986). Pharmacokinetics of (±)-, (+)-, and (−)-gossypol in humans and dogs. Clin. Pharmacol. Ther..

[25] Billiard J., Dennison J.B., Briand J., Annan R.S., Chai D., Colón M., Dodson C.S., Gilbert S.A., Greshock J., Jing J. (2013). Quinoline 3-sulfonamides inhibit lactate dehydrogenase A and reverse aerobic glycolysis in cancer cells. Cancer Metab..

[26] Olgiati K.L., Toscano W.A. (1983). Kinetics of gossypol inhibition of bovine lactate dehydrogenase X. Biochem. Biophys. Res. Commun..

[27] Caol S., Divers T., Crisman M., Chang Y.-F. (2017). In vitro susceptibility of Borrelia burgdorferi isolates to three antibiotics commonly used for treating equine Lyme disease. BMC Veter Res..

[28] Sicklinger M., Wienecke R., Neubert U. (2003). In Vitro Susceptibility Testing of Four Antibiotics against *Borrelia burgdorferi*: A Comparison of Results for the Three Genospecies *Borrelia afzelii*, *Borrelia garinii*, and *Borrelia burgdorferi* Sensu Stricto. J. Clin. Microbiol..

[29] Anderson M., Marayati R., Moffitt R., Yeh J.J. (2016). Hexokinase 2 promotes tumor growth and metastasis by regulating lactate production in pancreatic cancer. Oncotarget.

[30] Hamilton E., Fennell M., Stafford D.M. (1995). Modification of Tumour Glucose Metabolism for Therapeutic Benefit. Acta Oncol..

[31] Liu S., Kulp S.K., Sugimoto Y., Jiang J., Chang H.-L., Dowd M.K., Wan P., Lin Y.C. (2002). The (-)-enantiomer of gossypol possesses higher anticancer potency than racemic gossypol in human breast cancer. Anticancer Res..

[32] Dong Y., Mao B., Li L., Guan H., Su Y., Li X., Lian Q., Huang P., Ge R.-S. (2016). Gossypol enantiomers potently inhibit human placental 3β-hydroxysteroid dehydrogenase 1 and aromatase activities. Fitoterapia.

[33] Lordelo M.M., Davis A.J., Calhoun M.C., Dowd M.K., Dale N.M. (2005). Relative toxicity of gossypol enantiomers in broilers. Poult. Sci..

[34] Lindberg M.C., Naqvi R.H., Matlin S.A., Zhou R.H., Bialy G., Blye R.P. (1987). Comparative anti-fertility effects of gossypol enantiomers in male hamsters. Int. J. Androl..

[35] PubChem 3-[[3-(Cyclopropylsulfamoyl)-7-(2,4-dimethoxypyrimidin-5-yl)quinolin-4-yl]amino]-5-(3,5-difluorophenoxy)benzoic Acid. https://pubchem.ncbi.nlm.nih.gov/compound/71533725.

[36] Suzuki K., Maeda S., Morokuma K. (2019). Roles of Closed- and Open-Loop Conformations in Large-Scale Structural Transitions of l-Lactate Dehydrogenase. ACS Omega.

[37] Granchi C., Calvaresi E.C., Tuccinardi T., Paterni I., Macchia M., Martinelli A., Hergenrother P.J., Minutolo F. (2013). Assessing the differential action on cancer cells of LDH-A inhibitors based on the N-hydroxyindole-2-carboxylate (NHI) and malonic (Mal) scaffolds. Org. Biomol. Chem..

[38] Marlier J.F., Cleland W.W., Zeczycki T.N. (2013). Oxamate Is an Alternative Substrate for Pyruvate Carboxylase from *Rhizobium etli*. Biochemistry.

[39] Thornburg J.M., Nelson K.K., Clem B.F., Lane A.N., Arumugam S., Simmons A., Eaton J.W., Telang S., Chesney J. (2008). Targeting aspartate aminotransferase in breast cancer. Breast Cancer Res..

[40] Saleh S.R., Attia R., Ghareeb D.A. (2018). The Ameliorating Effect of Berberine-Rich Fraction against Gossypol-Induced Testicular Inflammation and Oxidative Stress. Oxidative Med. Cell. Longev..

[41] Santana A.T., Guelfi M., Medeiros H.C.D., A Tavares M., Bizerra P.F.V., Mingatto F.E. (2015). Mechanisms involved in reproductive damage caused by gossypol in rats and protective effects of vitamin E. Biol. Res..

[42] Ramsey M.E., Hyde J.A., Medina D.N., Lin T., Gao L., Lundt M.E., Li X., Norris S.J., Skare J.T., Hu L.T. (2017). A high-throughput genetic screen identifies previously uncharacterized Borrelia burgdorferi genes important for resistance against reactive oxygen and nitrogen species. PLoS Pathog..

[43] Bourret T.J., Boyle W.K., Zalud A.K., Valenzuela J.G., Oliveira F., Lopez J.E. (2018). The relapsing fever spirochete *Borrelia turicatae* persists in the highly oxidative environment of its soft-bodied tick vector. Cell. Microbiol..

[44] Radolf J.D., Samuels D.S. (2010). Borrelia: Molecular Biology, Host Interaction and Pathogenesis.

[45] Di Tommaso P., Moretti S., Xenarios I., Orobitg M., Montanyola A., Chang J.-M., Taly J.-F., Notredame C. (2011). T-Coffee: A web server for the multiple sequence alignment of protein and RNA sequences using structural information and homology extension. Nucleic Acids Res..

[46] Notredame C., Higgins D.G., Heringa J. (2000). T-coffee: A novel method for fast and accurate multiple sequence alignment. J. Mol. Biol..

