# Supplementary material for: Lactate Dehydrogenase Inhibitors Suppress Borrelia burgdorferi Growth In Vitro
Source: Pathogens. 2023 Jul 22;12(7):962. doi: 10.3390/pathogens12070962 (PMC10384987; doi:10.3390/pathogens12070962)

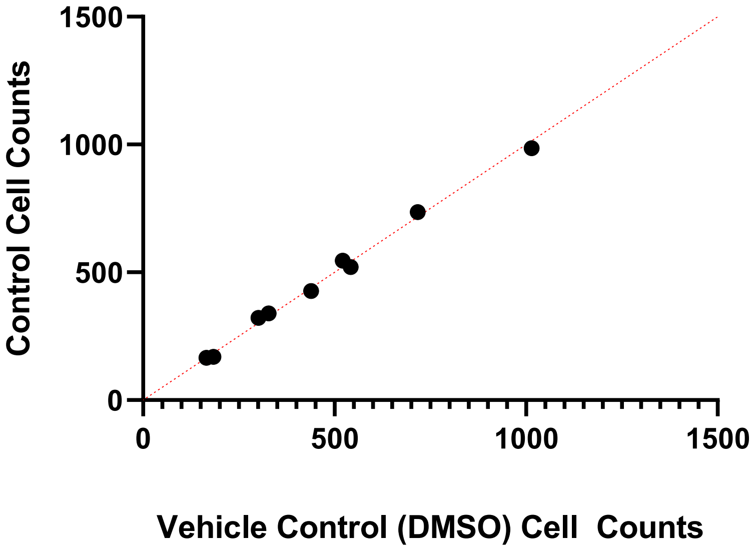

1500
1000
500
0
Control Cell Counts
0
500
1000
1500
Vehicle Control (DMSO) Cell Counts

Comparison of AT-101 and Gossypol impacts on Borrelia

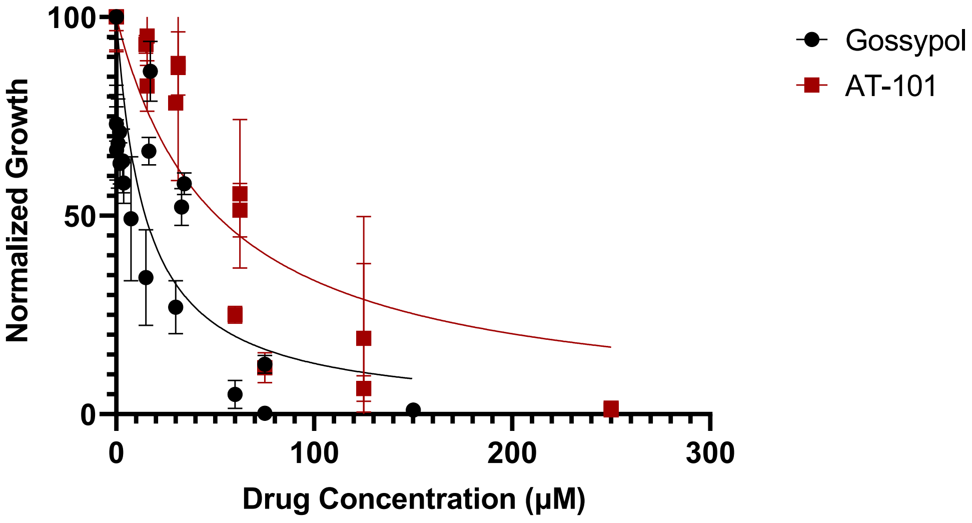

```
BbLDH        1 ML------KSNKVVLIGAGGVGSSFAYALTIDNSLVHELVIIDVNENKAKGEVMDLNHGQ
HsLDHA       1 MAQLIYNEPQNKITVVGVGAVGMACAISILM-KDLADELALVDVIEDKLKGEMMDLQHGS
consensus    1 * ......  **. ..* *.**    * . ..  *  ** ..** * * ***.***.**

BbLDH       55 MFLKKNINVLFGTYKDCANADIVVITAGLNQKPGETRLDLVDKNSKIFKDIITNVVSSGF
HsLDHA      60 LFLRTPKIVSGKDYNVTANSKLVIITAGARQQEGESRLNLVQRNVNIFKFIIPNVVKYSP
consensus   61 .**.    *    *   **  .*.****  *  **.** ** .*  *** ** ***

BbLDH      115 DGIFVVASNPVDIMTYVTMKYSKFPIHKVIGTGTILDTSRLRYFLSDHFNVNTQNIHSYI
HsLDHA     120 NCKLLIVSNPVDILTYVAWKISGFPKNRVIGSGCNLDSARFRYLMGERLGVHPLSCHGWV
consensus  121     .. ******.***  * * **  .***.*  **. * ** . ..  *     * ..

BbLDH      175 MGEHGDSSFATWDETKI---AMKPLSEYLAEGKITELELDEIHKKVVNAAYEVIKLKGAT
HsLDHA     180 LGEHGDSSVPVWSGMNVAGVSLKTLHPDLGTDKDKE-QWKEVHKQVVESAYEVIKLKGYT
consensus  181 .*******   *    .... .* *   *.  *  *.   *.** **  ********* *

BbLDH      232 YYAIGLGIKNIVNAIIGDQNVILPISSYINGQYGGLIKDIYIGAPAIVCKEGVKEVLNFK
HsLDHA     239 SWAIGLSVADLAESIMKNLRRVHPVSTMIKGLYG-IKDDVFLSVPCILGQNGISDLVKVT
consensus  241  .**** .  .   *.     . *.*. * * **..  *...  * *.   *. ...

BbLDH      292 ISPKELDKFNSSANQLKSYIDKMEF
HsLDHA     298 LTSEEEARLKKSADTLWGIQKELQF
consensus  301 ..  *  .   **  *      . *
```

A. Morphologies of Open vs. Closed form LDH

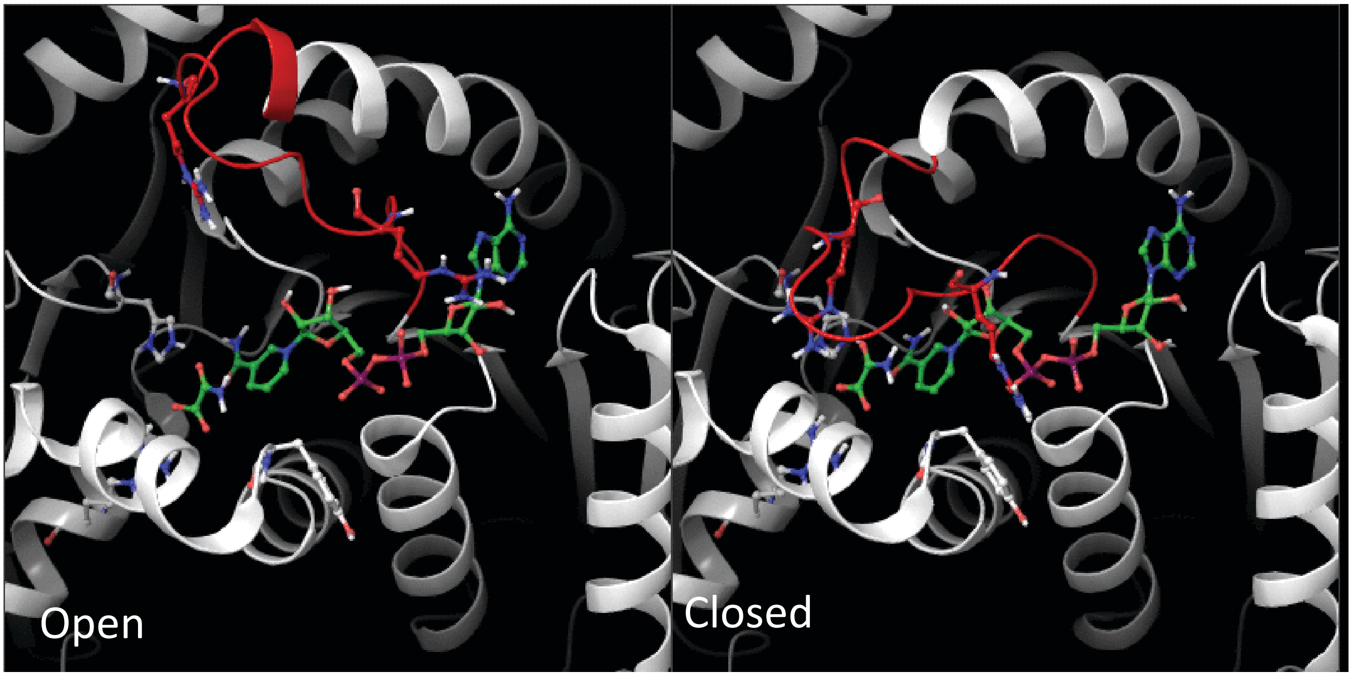

B. Gossypol targets the Open form of LDH

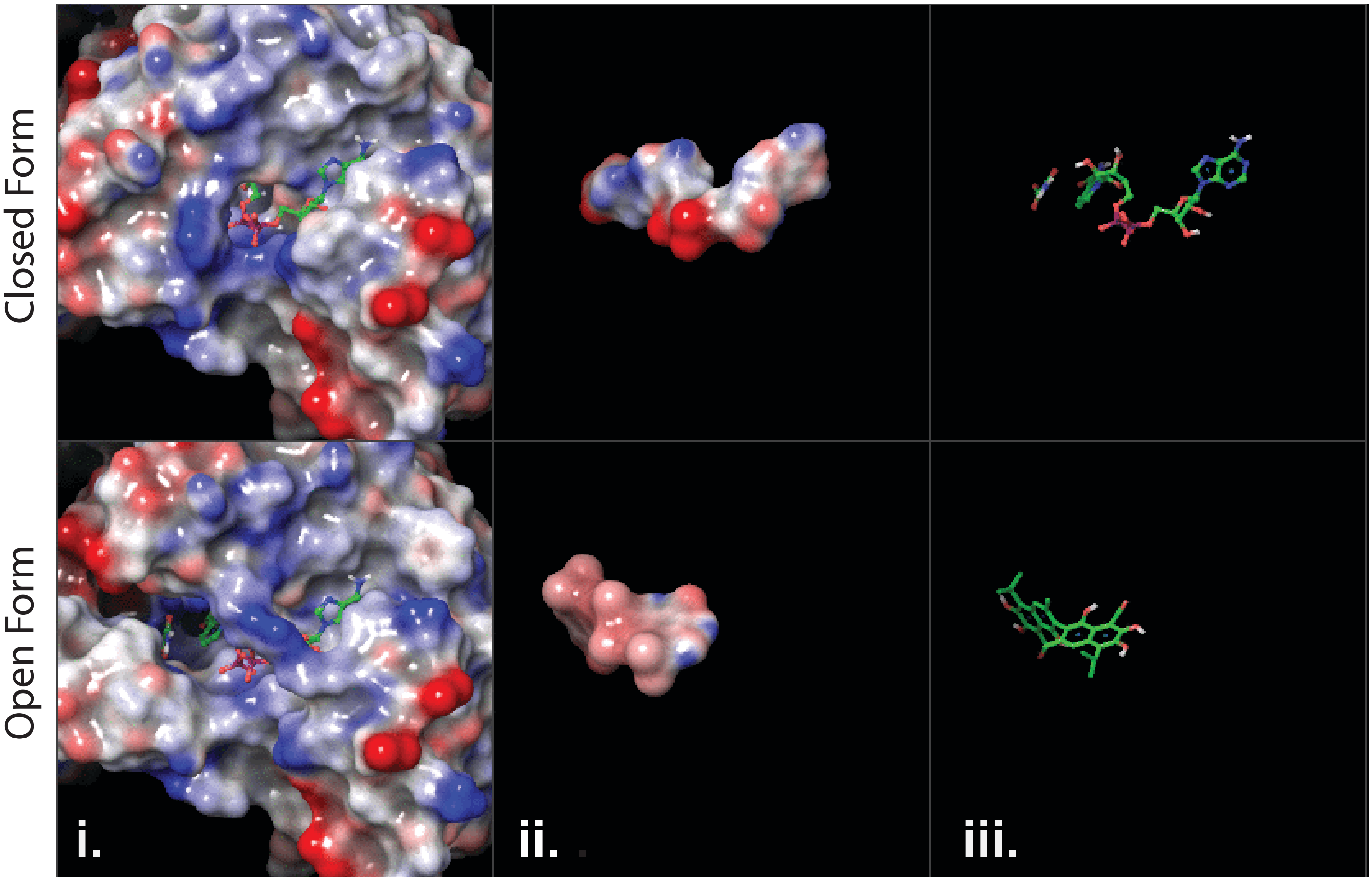

Supplement: Supplementary file 1 [file pathogens-12-00962-s001.zip › pathogens-2495785-supplementary.pdf]
